# Advancing Insights into Pediatric Macular Diseases: A Comprehensive Review

**DOI:** 10.3390/jcm14020614

**Published:** 2025-01-18

**Authors:** Lucia Ambrosio, Tatiana Perepelkina, Abdelrahman M. Elhusseiny, Anne B. Fulton, Jose Efren Gonzalez Monroy

**Affiliations:** 1 Department of Ophthalmology, University of Naples Federico II, 80138 Naples, Italy; 2 Department of Public Health, University of Naples Federico II, 80138 Naples, Italy; 3 Massachusetts Eye Associates, Chelmsford, MA 01824, USA; 4 Department of Ophthalmology, Harvey and Bernice Jones Eye Institute, Little Rock, AR 72205, USA; amelhusseiny@uams.edu; 5 Department of Ophthalmology, Boston Children’s Hospital, Boston, MA 02115, USA; 6 Department of Ophthalmology, Harvard Medical School, Boston, MA 02115, USA

**Keywords:** Stargardt disease, best disease, oculocutaneous albinism/foveal hypoplasia, X-linked juvenile retinoschisis

## Abstract

Pediatric macular disorders are a diverse group of inherited retinal diseases characterized by central vision loss due to dysfunction and degeneration of the macula, the region of the retina responsible for high-acuity vision. Common disorders in this category include Stargardt disease, Best vitelliform macular dystrophy, and X-linked retinoschisis. These conditions often manifest during childhood or adolescence, with symptoms such as progressive central vision loss, photophobia, and difficulty with fine visual tasks. Underlying mechanisms involve genetic mutations that disrupt photoreceptor and retinal pigment epithelium function, accumulating toxic byproducts, impaired ion channel activity, or structural degeneration. Advances in imaging modalities like optical coherence tomography and fundus autofluorescence have improved diagnostic accuracy and disease monitoring. Emerging therapies are transforming the treatment landscape. Gene therapy and genome editing hold promise for addressing the genetic basis of these disorders, while stem cell-based approaches and pharmacological interventions aim to restore retinal function and mitigate damage. Personalized medicine, driven by genomic sequencing, offers the potential for tailored interventions. Despite current challenges, ongoing research into molecular mechanisms, advanced imaging, and innovative therapies provides hope for improving outcomes and quality of life in children with macular disorders.

## 1. Introduction

Juvenile macular disorders represent a varied group of inherited retinal conditions that primarily affect the macula, the central part of the retina responsible for sharp vision. These disorders often arise during childhood or adolescence and are a significant contributor to early-onset vision loss [1]. Conditions within this group, such as Stargardt disease, Best vitelliform macular dystrophy, and juvenile X-linked retinoschisis, exhibit diverse genetic causes, distinct clinical symptoms, and varied pathological mechanisms, reflecting the complexity of these retinal diseases [2,3].

The primary pathological hallmark of macular juvenile disorders is the disruption of the photoreceptor–retinal pigment epithelium (RPE) complex, which plays a pivotal role in visual processing and photoreceptor homeostasis [4].

Clinically, juvenile macular disorders (Table 1) are characterized by a progressive decline in central vision, often accompanied by photophobia and difficulties with activities that require precise visual acuity, such as reading, recognizing faces, and discerning fine details [5]. Detailed fundoscopic examinations frequently reveal hallmark patterns specific to each disorder, including changes in pigmentation, deposits, or structural alterations in the macula. Recent advancements in imaging technologies, such as wide-field optical coherence tomography (OCT) and fundus autofluorescence (FAF), have significantly enhanced diagnostic accuracy and monitoring capabilities [6]. These non-invasive tools provide high-resolution, cross-sectional, and topographical views of the retina, allowing for detailed visualization of pathological changes, including disruptions in the retinal layers, accumulation of lipofuscin, and photoreceptor degeneration, thereby enabling early detection and precise disease progression tracking [7].

From a genetic perspective, macular juvenile disorders demonstrate Mendelian inheritance patterns, including autosomal dominant, autosomal recessive, and X-linked forms. Recent advances in genomic sequencing technologies, such as Next Generation Sequencing (NGS), have facilitated the identification of pathogenic variants and elucidated the molecular pathways implicated in these conditions.

NGS has revolutionized the field of genetics by enabling the rapid and comprehensive analysis of entire genomes or exomes. This advancement allows for the simultaneous sequencing of millions of DNA fragments, providing detailed insights into genetic variations and their roles in health and disease. In clinical settings, NGS has transformed approaches to diagnosing and managing genetic disorders. Its capacity for massive parallel sequencing facilitates personalized precision medicine, enabling tailored therapeutic decisions based on an individual’s genetic profile [8,9,10,11].

The impact of NGS is evident in rare genetic disorders, where it assists in uncovering previously elusive diagnoses [12]. As NGS technology continues to evolve, it holds the promise of further enhancing our understanding of complex genetic diseases and improving patient care through more precise and individualized treatment strategies. These insights have paved the way for the development of targeted therapeutic approaches, including gene therapy, pharmacological interventions aimed at reducing toxic metabolite accumulation, and retinal prosthetics to restore visual function [13,14,15].

The study of macular juvenile disorders not only enhances our understanding of retinal biology but also holds promise for developing innovative treatments to mitigate visual impairment in affected individuals. As research progresses, multidisciplinary approaches integrating genetics, molecular biology, and clinical ophthalmology will be essential to address the challenges posed by these complex diseases.

## 2. Relevant Sections

### 2.1. Stargardt Disease

The most common form of inherited macular dystrophy is Stargardt disease (STGD1), also called juvenile macular dystrophy or fundus flavimaculatus. It affects approximately 1 in 8000 to 1 in 10,000 people and is caused by mutations in the *ABCA4* gene [16,17]. First described in 1909 [17] by German ophthalmologist Karl Stargardt, the condition is characterized by significant phenotypic and genotypic variability. With the exception of foveal-sparing forms, Stargardt disease typically leads to progressive retinal degeneration and vision loss in children and young adults, imposing considerable socioeconomic and psychological challenges.

STGD1, resulting from mutations in the *ABCA4* gene, which encodes a photoreceptor-specific ATP-binding cassette subfamily A member 4 transporter, follows an autosomal recessive inheritance pattern. The ABCA4 gene, consisting of 50 exons and specific to photoreceptors, plays a key role in Stargardt disease. Additionally, mutations in the EVOL4 and PROM1 genes have been associated with autosomal dominant forms of Stargardt-like dystrophies [18]. Extensive genetic analyses have identified over 900 pathogenic variants to date [19,20]. This genetic diversity contributes to substantial clinical variability among STGD1 patients, including differences in age of onset, progression rate, and disease severity. Establishing genotype–phenotype correlations remains a significant challenge.

The ABCA4 gene produces an ATP-binding cassette transporter that resides on the edges of photoreceptor outer segment disks in both rods and cones. This protein is vital for managing vitamin A byproducts, particularly by transporting N-retinylidene-phosphatidylethanolamine (N-RPE), a byproduct of 11-cis-retinal generated during the early stages of phototransduction. Once transported from the disk space into the cytoplasm, N-RPE is reduced and then processed by the retinal pigment epithelium (RPE) to regenerate 11-cis-retinal. When the ABCA4 protein fails to function properly, N-RPE builds up, giving rise to toxic substances like lipofuscin and A2E, which can severely damage RPE cells. Since RPE cells and photoreceptors are metabolically interdependent, damage or destruction of RPE cells ultimately results in photoreceptor death [20].

A thorough review of the history of vision symptom development is essential, although early symptoms of juvenile macular dystrophy are often variable and nonspecific. In our cohort of 54 patients, the onset of symptoms occurred between the ages of 7 and 10. Similarly, the Progression of Atrophy Secondary to Stargardt Disease (ProgStar) study, a multicenter, prospective cohort study, aimed at understanding the natural progression of STGD1. Its primary objective was to identify reliable outcome measures for future clinical trials and to enhance the understanding of disease progression over time. The study enrolled 233 individuals with genetically confirmed STGD1, who were monitored over a 24-month period with evaluations every 6 months. Key assessments included microperimetry to measure macular function and retinal sensitivity, providing insights into the rate of functional decline in patients. The data collected have facilitated the development of new volumetric visual function indices and have informed the design of interventional clinical trials aimed at slowing or halting the progression of Stargardt disease. ProgStar reported a mean age of symptom onset of 7.2 years in 51 patients with early-onset Stargardt disease [16]. By this age, children can usually express challenges with their vision, and many are diagnosed after not passing school vision screenings. In addition to documenting the onset and progression of symptoms, gathering information about prior medical care and any family history of visual problems is crucial. Retrieving previous medical records, when available, can also provide valuable insights.

Common initial visual complaints include failed vision screenings at school or during pediatric visits, difficulty seeing the board, trouble reading, and abnormal visual behaviors noted by teachers or parents, such as maintaining a very short distance from the TV or computer, holding objects very close to the face, or failing to notice objects like a hockey puck. Additionally, a lack of improvement with glasses and abnormal eye movements are often observed. Older children (ages 10–12) may describe specific visual disturbances, including blurriness in their visual field, central scotomas, or dots appearing on a light surface.

Misdiagnosed STGD1 can sometimes lead to significant frustration in children due to lagging behind peers in school or kindergarten, declining reading levels, and the development of unsettling psychological, behavioral, and motor issues. These challenges are often compounded by diagnoses such as attention-deficit/hyperactivity disorder (ADHD) or dyslexia.

Younger siblings of children diagnosed with STGD1 are frequently brought in for an assessment at a very young age, sometimes during babyhood. We recommend offering genetic testing to these siblings and closely monitoring them for symptom development. Early identification not only enables timely intervention but also enhances awareness of the disease’s progression.

A thorough pediatric ophthalmic examination should be conducted, incorporating age- and developmentally appropriate vision testing, stereopsis, assessment of eye alignment, slit lamp examination, perimetry (if age-appropriate), ophthalmoscopy, OCT, and cyclorefraction.

In the early stages of Stargardt disease, ophthalmoscopy may reveal a normal-appearing fundus. Due to the heterogeneity of Stargardt disease, providing a universal description of physical findings is difficult and often relies on imaging and ancillary studies. This challenge is compounded by the variability in the disease’s progression timeline. Best corrected visual acuity (BCVA) can range from 20/30 in the early stages to as poor as 20/600 in the late stages. According to the ProgStar study, the median time to develop severe visual impairment (BCVA worse than 20/200) was approximately 12 years [16,21]. The majority of children with STGD1 experience moderate to severe visual impairment when classified by visual acuity alone.

**Figure 1:**
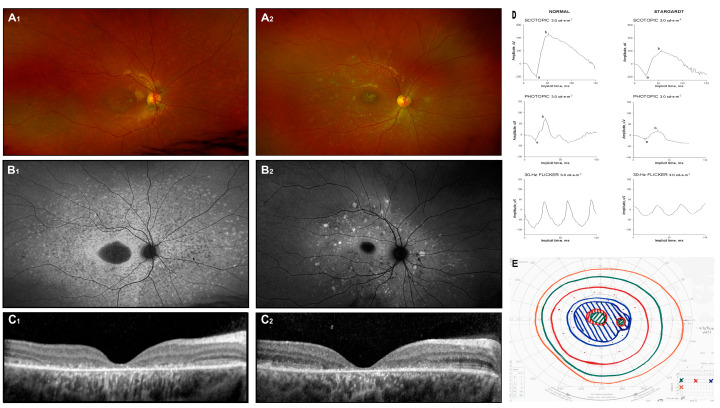
Stargardt disease. Fundus image (**A_1_**,**A_2_**), autofluorescence image (**B_1_**,**B_2_**), and OCT scans (**C_1_**,**C_2_**) of two unrelated patients with biallelic ABCA4 mutations. Symptoms appeared around 6 years of age, and the images were captured at approximately 12 years (**D**,**E**). Additionally, the ERG results (a: a-wave; b: b-wave) and Goldman perimetry outcomes for patient 1 at 12 years of age are presented.

Distinctive findings on ophthalmoscopy exams include foveal thinning and yellow deposits, or “flecks”, at the posterior pole of the retina (Figure 1(A_1_,A_2_)). Ocular misalignment may not be evident in the early stages but is commonly observed later as the disease progresses. Advanced stages, characterized by macular atrophy and the enlargement of central scotoma, often result in inconsistent and off-center fixation [22].

Diagnosis is supported by the use of fundus photography, FAF imaging, spectral-domain optical coherence tomography (SD-OCT), and full-field electroretinography (ffERG), which are essential diagnostic tools. Color fundus photographs typically reveal atrophic lesions, pigmentary changes, and characteristic flecks (Figure 1(A_1_,A_2_)). On corresponding FAF images, macular lesions are characterized by decreased autofluorescence, indicating areas of atrophy. Active flecks, associated with lipofuscin accumulation, appear as hyperfluorescent deposits, while older flecks, indicative of RPE atrophy and photoreceptor impairment, look hypofluorescent (Figure 1(B_1_,B_2_)) [23].

Macular SD-OCT often shows progressive disruption of the photoreceptors’ inner segment layer and thinning of the outer nuclear layer (Figure 1(C_1_,C_2_)). However, in the early stages of the disease, these findings are typically absent, and the severity and distribution of lesions vary significantly among individuals. Fishman et al. (1976) described the progressive changes in phenotype [24], while Cukras et al. (2012) observed a centrifugal progression of flecks. This pattern distribution may reflect underlying molecular mechanisms driving the disease process [23].

Electroretinographic (ERG) responses in Stargardt disease can exhibit a wide spectrum of deviations. Researchers categorize patients according to ERG findings, grouping them into classifications such as normal, mild, or severe dysfunction (Ref. [25]) or into groups with normal dark-adapted but reduced light-adapted responses, or both dark-adapted and light-adapted abnormalities [26]. Despite these efforts, no consistent correlation has been established between ERG findings, specific mutations, and fundus appearance. Example ERG testing is shown in Figure 1D: responses are attenuated in all stimulus conditions (scotopic 3.0, photopic 3.0, and flicker 30 Hz) (Ref. [27]). None of the mentioned classification systems—whether derived from fundoscopic appearances or ERG outcomes—has gained widespread acceptance in clinical practice.

Differentiating Stargardt disease from other cone–rod dystrophies is crucial, with a definitive molecular identification relying on genetic testing. In cases where fundoscopic changes are absent, early diagnosis can be particularly challenging. Among our cohort of STGD1 patients, the average delay between the onset of symptoms and a confirmed diagnosis was approximately three years. Establishing a diagnosis is critical for both families and clinicians, as it allows for appropriate monitoring, counseling, and disease management.

Throughout the natural history of the disease, BCVA generally deteriorates gradually. However, some reports indicate that vision may stabilize at approximately 20/200 [17]. In later stages, central scotoma (Figure 1E) and eccentric fixation are commonly observed.

Given the lack of effective treatments, management mainly involves tracking disease progression and offering appropriate support for educational and/or professional activities, such as magnification devices, screen-reading applications, and enhanced vision devices for TVs and computers. Support should also include social and psychological services, as well as occupational counseling. Consultation with a low-vision specialist is recommended, and patients should be encouraged to consider learning Braille and utilizing orientation and mobility services. Genetic counseling is also advised.

There is currently no definitive treatment available for Stargardt disease, but several potential strategies are under investigation, with some already advancing to clinical trials. These approaches include pharmacologic treatments, gene therapy, and stem cells.

The pharmacologic strategies, which primarily aim to reduce toxic retinoids, include the following:

Emixustat hydrochloride: This drug inhibits RPE65 and reduces the production of 11-cis-retinal (NCT03772665, NCT03033108). Preliminary results suggest biological activity in STGD1 patients [28]. Dose-dependent suppression of rod b-wave amplitude recovery post photobleaching confirms emixustat’s biological activity. However, no meaningful differences regarding macular atrophy have been demonstrated between treatment groups.

STG-001 (Stargazer Pharmaceuticals): This treatment lowers the plasma concentration of retinol-binding protein (RBP4), thereby reducing vitamin A absorption and retinoid accumulation (NCT04489511, NCT05244304, NCT05266014).

ALK-001 (Alkeus Pharmaceuticals): A chemical deuterated form of vitamin A designed to substitute for regular vitamin A, reducing the formation of retinoids (NCT02402660, NCT04239625, NCT02230228).

Inhibition of visual cycle (4-Methylpyrazole): No effect on dark adaptation has been demonstrated in healthy probands; further studies are suspended because the substance does not seem to inhibit the visual cycle strongly enough (NCT00346853).

Additionally, complement-mediated therapies for STGD1, similar to those being explored for age-related macular degeneration (AMD) and geographic atrophy, are also under investigation, such as the complement C5 inhibitor (NCT03364153).

Clinical trials exploring the efficacy of supplements such as Omega-3 Fatty Acids, Docosahexaenoic acid (DHA), and Saffron demonstrated modest efficacy (NCT03297515, NCT00420602, NCT00060749, NCT01278277).

Genetic replacement therapy trials were terminated, due to a lack of efficacy. However, the participants who received subretinal gene replacement therapy are still being monitored (NCT01367444, NCT01736592). Treatment was well tolerated, but no clinically significant changes in visual function tests were found [29].

Optogenetics is a genetic therapy designed for advanced disease stages, where residual non-photoreceptor cells are rendered light-sensitive through the use of AAV vectors to deliver an opsin-related photopigment. This strategy is currently under investigation in a Phase II clinical trial for STGD1, utilizing AAV2 to deliver a multicharacteristic opsin gene expression cassette (NCT05417126).

For the treatment of advanced disease, cell replacement strategies present a promising avenue. A completed Phase I/II clinical trial (NCT01469832) investigating human embryonic stem cell (hESC)-derived RPE cells for severe advanced STGD1 reported subretinal hyperpigmentation at the United Kingdom trial site, indicative of the survival of transplanted hESC-derived RPE cells [30]. Modest improvements in BCVA were observed. Microperimetry did not reveal significant functional benefits at 12 months [31]. A Phase I trial (NCT01625559) assessing the long-term safety and tolerability of hESC-derived RPE cells reported no adverse events and yielded favorable outcomes [32]. Future studies are anticipated, including trials exploring combined RPE and photoreceptor transplantation derived from either hESCs or induced pluripotent stem cells (iPSCs).

Additionally, trials employing autologous bone marrow-derived stem cells (BMSCs) are progressing at different stages. One study (NCT01920867) demonstrated visual acuity improvement in 61.8% of treated eyes, with 76.9% of patients experiencing overall visual improvement. Another BMSC study (NCT03011541) is ongoing, with results yet to be published.

### 2.2. Best Disease (Juvenile Vitelliform Macular Dystrophy)

Best vitelliform macular dystrophy (BVMD), also referred to as Best disease, is a progressive retinal dystrophy with early manifestation. It is the most frequent among the five distinct degenerative disorders linked to mutations in the *BEST1* (VMD2) gene, collectively known as “bestrophinopathies” (Ref. [33]). The condition is named after Friedrich Best, who first described a family with this macular dystrophy [34]. While the exact prevalence is not well established, most studies focus on Caucasian or European populations. Earlier estimates of Best disease incidence include 2 in 10,000 individuals in Sweden and 1.5 in 100,000 in Denmark. A recent study conducted in Minnesota marked the first report of BVMD prevalence in the United States, revealing an occurrence rate of 1 in 16,500 to 1 in 21,000 in that population [34,35,36,37].

BVMD is primarily inherited in an autosomal dominant pattern, though incomplete penetrance and a broad phenotype can be seen [38]. The gene is located on chromosome 11q12-13 and consists of 11 exons. Over 200 mutations in the *VMD2* gene have been identified up to now, with some sources suggesting the total number could be as high as 300 [39,40]. *VMD2* is exclusively expressed in the RPE, which plays a crucial role in retinal development and preservation. Mullins et al. showed that bestrophin expression is higher in the regions of the retina outside the macula [41]. This uneven expression supports the hypothesis that, in the case of BEST disease, peripheral RPE can compensate and function normally with one functioning copy of the *VMD2* gene. However, in the macula, the RPE becomes insufficient, leading to the characteristic macular lesions. Alternatively, the unique structures of the foveal and parafoveal regions may contribute to the presence of macular lesions [41].

BEST1 encodes bestrophin, an integral transmembrane protein with a complex mechanism of action. It is a calcium-dependent chloride and bicarbonate channel that regulates ion transportation. Additionally, it serves as an inhibitor of voltage-dependent intracellular calcium channels. Data suggest that *BEST1* plays a part in ocular maturity. Although the precise mechanism in ocular development is not fully understood, specific mutations in *VMD2* have been associated with ocular abnormalities involving the cornea, crystallin, and retina [39,42,43]. The current understanding of the pathophysiology can be summarized as follows: mutations in bestrophin disrupt ion and fluid transport, leading to RPE dysfunction. This impairs the RPE microvilli and their interaction with photoreceptor outer segments, resulting in serous retinal detachment. Furthermore, compromised phagocytosis causes the subretinal accumulation of lipofuscin, which contributes to the characteristic vitelliform or “egg-yolk” appearance [39,40].

The majority of affected patients have a meaningful family record. Affected patients, aware of the hereditary nature of the disease, may take their child for an eye exam before symptoms appear. However, the absence of other affected members of the family does not rule out the diagnosis, considering the variation in phenotype, age of onset, and the fact that 7–9% of individuals carrying *BEST1* mutations may have visual testing within the normal limits [33]. The disorder can also result from a de novo mutation [34,44,45]. In some cases, the clinical diagnosis may remain invisible until adulthood, particularly if visual acuity is only minimally impaired. Onset typically occurs before puberty, most often between the ages of 3 and 15, with an average age of 6 years reported in some studies [39,44]. In a recent natural history report, among the three most common variants, p.(Ala243Val) was associated with a later age of onset, better age-adjusted BCVA, and less advanced Gass stages compared with p.(Arg218Cys) and p.(Arg218His) [46].

The primary complaint at the ophthalmologic evaluation is typically reduced or distorted vision. Patients may also report metamorphopsia and central vision deficits. Although the primary clinical features are distinctly localized at the posterior pole, visual acuity may still be normal at the time of diagnosis. Patients often experience metamorphopsia and central vision impairments. The disease frequently progresses asymmetrically, with different stages present in each eye (as detailed in the clinical stages below) [39,47].

Visual acuity varies significantly depending on the disease stage, ranging from 20/20 to 20/200, and may worsen if complications develop. Notably, there is a strong correlation between visual acuity and factors such as age, disease stage, and the size of the central scotoma [47,48].

**Figure 2:**
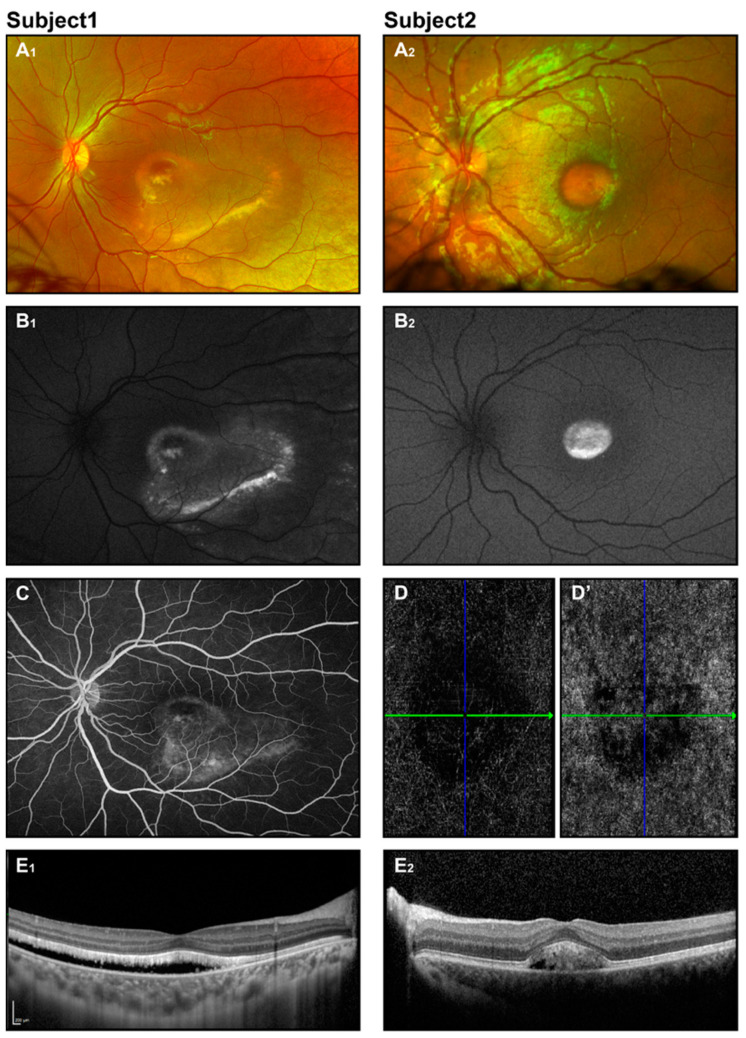
Best disease. Retinal imaging of two subjects (ages 8 and 6 years) with Best disease. Fundus photos display vitelliform macular lesions (**A_1_**,**A_2_**), with enhanced signal on corresponding FAF images (**B_1_**,**B_2_**), suggesting the vitelliruptive and pseudohypopyon stages for subjects 1 and 2, respectively. Fluorescein angiography for subject 1 (**C**) revealed no leakage. OCT angiography for subject 2 (**D**,**D’**) showed an avascular choriocapillaris layer and no evidence of CNV. OCT scans (**E_1_**,**E_2_**) depict neuroretinal detachment.

A detailed pediatric eye assessment should be carried out, including age-appropriate tests for visual acuity, evaluation of depth perception and binocular vision, assessment of eye movement and disalignment, inspection of the anterior segment, perimetry, ophthalmoscopy, and cycloplegic refraction. Strabismus or significant refractive errors may impact treatment decisions. Anterior chamber abnormalities have been reported in association with Best disease. Wittstrom et al. [49] described three adults and one child with BVMD, with small to moderate hyperopia in adults and high hyperopia in a 10-year-old, along with shorter axial lengths. Axial hyperopia is a known risk factor for angle-closure glaucoma [50]. The hallmark finding in the fundus exam is the “egg-yolk” macular lesion, which may be solitary or multifocal [51]. These lesions are well circumscribed, circular, yellow-opaque patches ranging from 0.5 to 2 disc diameters in size (Figure 2(A_1_,A_2_)).

Best vitelliform dystrophy is classified into five clinical stages [33,49,52,53]:

Previtelliform—Normal vision and fundus appearance, with subtle RPE alterations.

Vitelliform—Presence of vitelliform “egg-yolk” lesions, with normal or mildly decreased vision.

Pseudohypopyon—Stratification of accumulated material with no significant changes in vision.

Vitelliruptive—Disturbance or partial breakdown of material, giving a “scrambled egg” appearance; vision worsens.

Atrophic—Chorioretinal atrophy and scarring, with markedly reduced vision (20/200).

Deutman [54], Mohlerand Fine [55], and Gass [56] have proposed similar classifications. However, Gass positions the pseudohypopyon stage before the vitelliruptive stage (stages III and IV). In contrast, Clemett [57] suggested that this clinical sequence might be reversed and that these stages can alternate over the disease course. When the vitelliform material breaks, a horizontal fluid level appears [34,39]. Additionally, neovascularization and leakage are sometimes considered either part of stage V or a separate stage VI, occurring in 2–9% of Best dystrophy cases [39].

Additionally, many patients present with different stages in each eye [57], and many BVMD lesions exhibit features characteristic of multiple BVMD stages simultaneously [38]. Remarkably, even eyes with lesions in the cicatricial stage often maintain good visual acuity despite a significant central scarring appearance [55,58].

Imaging plays a crucial role in the early and accurate diagnosis of Best vitelliform dystrophy. Fundus photographs, fundus autofluorescence, and OCT images are typically obtained during a retinal evaluation when age- and medically appropriate for the child.

Fundus photography reveals classical macular lesions or may show a normal fundus appearance in the early stages (Figure 2(A_1_,A_2_)). In OCT, neuroretinal separation is seen with hyperreflective deposits and hyporeflective fluid situated between the photoreceptors and the retinal pigment epithelium (RPE). Atrophic and fibrotic regions manifest as pronounced hyperreflective areas [33,35,39] (Figure 2(E_1_,E_2_)).

Fundus autofluorescence imaging shows a pronounced increase in autofluorescence signal in the vitelliform lesion. As the disease progresses, the autofluorescent pattern evolves, with granulation seen in the vitelliruptive stage and a decrease in or absence of the signal in the atrophic stage (Figure 2(B_1_,B_2_)). In contrast, fluorescein angiography (FA) exhibits a pattern that starts with hypofluorescence in the area of the lesion, evolving to a lumpy appearance, and finally showing hyperfluorescence in the atrophic stage or in the presence of neovascularization and leakage (Figure 2C) [34,39]. Since intravenous dye administration may be difficult in some patients, FA may be performed under anesthesia.

OCT angiography (OCT-A), a non-invasive technique, provides excellent visualization of retinal and choroidal flow and is considered by some to be an effective or even superior alternative to FA. Abnormal findings involve distortion of the foveal avascular zone, loss of retinal vascularity, and different patterns of choroidal neovascularization [59,60].

Historically, an abnormal electrooculogram (EOG) was used as a diagnostic criterion for BVMD. The hallmark feature of Best dystrophy is a low ratio of potentials recorded between the cornea and retina (Arden ratio < 1.55). The EOG is abnormal across all phases of the disease, while the ERG is typically normal or nearly so [33,38,39]. With the widespread use of genetic testing, electrophysiologic tests have become less essential for diagnosis.

Although the fundus appearance of BVMD, along with family history, is often distinctive, the differential diagnosis should consider other conditions, including central serous chorioretinopathy, infectious retinochoroiditis, Stargardt disease, and other retinal degenerations. Ultimately, a definitive diagnosis is usually confirmed through molecular testing.

Currently, there is no resolutive treatment for BVMD. However, gene therapy and stem cell-based approaches are being explored. Complications can be managed. For example, choroidal neovascularization can be treated with intravitreal anti-vascular endothelial growth factor (VEGF) injections, laser photocoagulation, or photodynamic therapy [50,61,62,63,64]. In a recent review, eyes with choroidal neovascularization that received treatment with an anti-VEGF agent showed better mean BCVA compared with eyes that were not treated with an anti-VEGF agent [46]. A rare complication, such as a macular hole with or without detachment, is typically treated surgically [65,66,67].

The visual prognosis for BVMD is generally favorable. According to some sources, the best corrected visual acuity ranges from 20/20 in the previtelliform stage to 20/200 or worse in the atrophic and choroidal neovascularization stages [34,68].

### 2.3. Oculocutaneous Albinism/Foveal Hypoplasia

Albinism, originating from the Latin word *albus,* for “white”, describes a collection of genetically varied conditions that impact the production of melanin. In Western populations, its occurrence is estimated to fall between 1 in 17,000 and 1 in 20,000 individuals [69]. Albinism is categorized into two main types: oculocutaneous albinism (OCA), which affects the skin, hair, and eyes, and ocular albinism (OA), which involves only the eyes. Common ocular features include reduced visual acuity, retinal hypopigmentation, iris translucency, photophobia, foveal hypoplasia, nystagmus, abnormal brain connections (with atypical decussation of optic nerves at the chiasm), and eye misalignment [69,70,71]. A recent study on 50 patients with albinism showed a correlation among nystagmus type, visual acuity, and grades of foveal hypoplasia. They demonstrated that foveal hypoplasia grade 4 is associated with pendular nystagmus, a shorter foveation period, and worse visual acuity [72].

There are seven known genes linked to seven distinct types of isolated oculocutaneous albinism, inherited as autosomal recessive. Four of these (OCA1–OCA4) are associated with the genes *TYR*, *OCA2*, *TYRP1*, and *SLC45A2*. The most severe form, OCA1A, is caused by two null mutations in the *TYR* gene, leading to complete tyrosinase inactivity and a lifelong absence of melanin production. Milder variants of OCA allow for some melanin accumulation over time [73,74,75,76]. Despite over 500 documented mutations, some cases of OCA remain genetically unresolved [74].

In contrast, OA involves a single gene, *GPR143*, and is inherited in an X-linked recessive manner [71,76].

In summary, mutations causing albinism lead to impaired melanin production. For instance, the *TYR* gene encodes tyrosinase, an enzyme involved in melanin biosynthesis that converts L-tyrosine to L-DOPA and then to dopaquinone. The *OCA2* gene is responsible for producing the P-protein, an integral melanosomal protein essential for constructing melanosomes—organelles responsible for pigment production. *TYRP1* encodes tyrosinase-related protein-1, which stabilizes tyrosinase; mutations in this gene delay tyrosinase maturation and promote early degradation. The *SLC45A2* gene encodes a protein believed to function as a membrane transporter within melanosomes [74,77]. In ocular albinism, the *GPR143* gene encodes a G-protein-coupled receptor critical for melanosome development and maturation [78].

Foveal hypoplasia is a shared characteristic across all forms of OCA. The mechanisms underlying the lack of foveal development remain unclear. Melanin is known to play a role in regulating the development of both ocular structures and visual pathways, including the abnormal projection of some temporal retina fibers to the contralateral visual cortex in albinism. However, the exact mechanisms responsible for these anomalies are not fully understood [70,79,80]. Studies have investigated how melanin affects the spatial–temporal development of retinal ganglion cells (RGCs) and optic pathways. Impaired melanin synthesis is thought to impact the timing of RGC generation, development, and the establishment of ipsilateral projections [79,80,81,82,83].

Research on foveal cone specialization in albinism has yielded mixed findings. While there is no consensus on albino foveal development, studies report varying degrees of foveal maturation, with some describing decreased central cone density and others noting increased densities [84,85,86].

The range of physical characteristics across various forms of albinism is considerable, with the easily recognizable skin-related traits observed in OCA1A, which families often easily identify. People with OCA1A usually exhibit completely white hair, eyelashes, and skin, along with pink-colored eyes. In cases of albinism where pigmentation is partially retained or in populations with naturally lighter skin tones, assessing differences relative to other relatives can be essential for diagnosis [87,88].

The most concerning symptom for parents of children with albinism is often nystagmus. Consequently, many patients initially present to neurology or neuro-ophthalmology. It is important to obtain a detailed history and description of the nystagmus. In many cases, an MRI is recommended to assess for related brain anomalies [88].The primary concerns leading to an office visit are often nystagmus, reduced vision, and photophobia. Nystagmus typically appears within the first few weeks of life and tends to diminish over time. Visual acuity gradually improves with age, generally correlating with the degree of pigmentation. As nystagmus subsides, visual acuity often shows further improvement [70].

Visual acuity for different types of OCA typically varies: very low acuities for OCA1A, to 20/80, and to 20/100 for OCA2 [86].

A complete ophthalmic evaluation is recommended. Nystagmus, often accompanied by compensatory head posturing, is commonly observed. Children with OCA exhibit an increased incidence of strabismus, which may be associated with an abnormal chiasm anatomy [70]. In addition to varying degrees of reduced pigmentation, decreased iris pigment is a notable feature. Areas of the iris that transilluminate light may range from subtle to pronounced, with severe cases allowing the periphery of the eye lens to become detectable. A slit-lamp exam should be performed whenever feasible.

**Figure 3:**
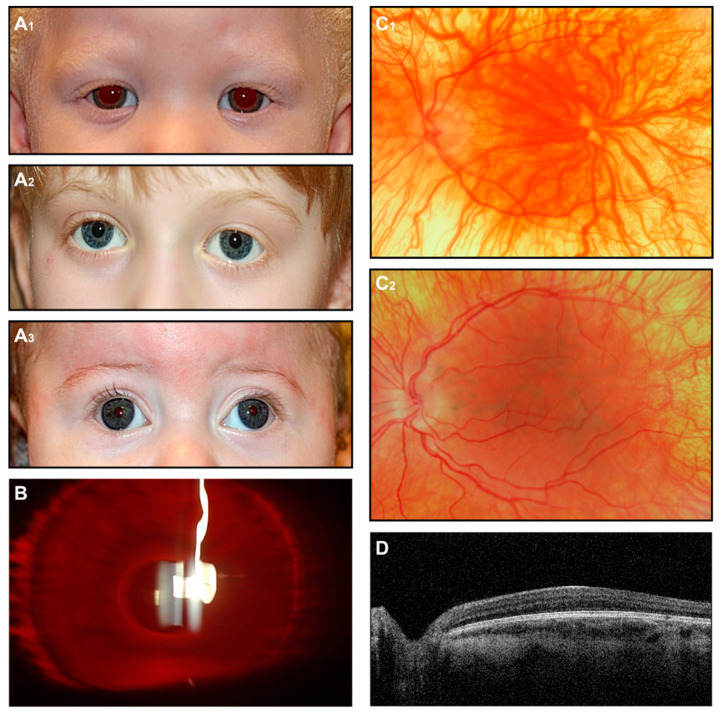
OCA. (**A**) External images of individuals diagnosed clinically with OCA (**A_1_**), molecularly confirmed OCA2 (**A_2_**), and OCA4 (**A_3_**). (**B**) Slit-lamp image of the OCA subject shows iris transillumination. (**C_1_**,**C_2_**) Fundus images of two subjects with a clinical diagnosis of OCA. (**D**) OCT scan shows foveal hypoplasia.

Anterior segment anomalies, including findings such as posterior embryotoxon, Axenfeld anomaly, and aniridia, have been reported in syndromes associated with foveal hypoplasia [89,90]. Ophthalmoscopic examination typically reveals a lack of a foveal reflex, along with a translucent, yellowish retina and prominent visualization of the choroidal vessels (Figure 3).

When physical examination findings clearly indicate a form of albinism, additional imaging is generally not necessary for diagnosis. Nevertheless, fundus photography and, in particular, OCT can be performed to provide proof in uncertain individuals. This is particularly valuable in OCA individuals with some preserved pigmentation, where the clinical presentation may not strongly suggest albinism. Although OCT can be challenging to perform in young patients with nystagmus, it can reveal characteristic features such as foveal hypoplasia and preserved inner retinal layers, with variable degrees of disrupted foveal architecture reported [71,74].

Full-field ERG testing, if obtained, is typically normal, although cases of above-average amplitudes have been previously documented [91,92].

The diagnosis of albinism is typically based on characteristic skin and ocular findings, with confirmation through genetic testing. It is important to distinguish OCA from syndromes associated with melanin production defects, including the following [69,77,87,93]:

Chediak–Higashi Syndrome: A condition that causes recurrent infections, coagulation abnormalities, neurological impairment, and hypopigmentation.

Hermansky–Pudlak Syndrome (HPS): Although rare, HPS has notable global occurrence, particularly among Swiss and Puerto Rican populations. Key features include bleeding tendencies, neutropenia, pulmonary fibrosis, and granulomatous colitis.

Waardenburg Syndrome: An autosomal dominant disorder caused by mutations in the *MITF* gene. Symptoms include sensorineural hearing loss, hypopigmented skin patches, and iris heterochromia or stromal atrophy.

Griscelli Syndrome: A rare condition marked by hypopigmented skin, silver-gray hair, and, in some cases, neurological malformations.

Tietz Albinism–Deafness Syndrome: An autosomal dominant disorder linked to the *MITF* gene, presenting with sensorineural hearing loss and hypopigmented fundi.

Angelman and Prader–Willi Syndromes: These neurodevelopmental disorders, caused by maternal or paternal microdeletions in chromosome 15q11-13, can be associated with hypopigmentation.

Other forms of infantile nystagmus or other inherited retinal dystrophies, including Achromatopsia, should be considered in the differential diagnosis.

Currently, there is no treatment for albinism. Preclinical studies have shown promising results with nitisinone, an FDA-approved drug for tyrosinemia, which was found to enhance fur and ocular pigmentation in mouse models by inhibiting tyrosine degradation. However, a one-year pilot study in humans failed to demonstrate increased melanin in skin, hair, or iris pigmentation [94].

Another investigational treatment, L-DOPA, was evaluated in a randomized trial involving 45 patients, but no improvement in visual acuity was observed [69]. Therapeutic trials involving L-DOPA and nitisinone aimed to restore retinal L-DOPA levels. Most studies focused on adult participants, where foveal maturation is largely complete. No significant treatment effects were observed in humans, and no major safety concerns were identified in the trials. Future research could explore perinatal administration, although this approach would require careful consideration of ethical and safety issues. Additionally, administering zeaxanthin and lutein to patients with albinism has not demonstrated any observable effects. The potential for gene therapy is also under investigation as a future treatment option [95].

Albinism is a static condition with a good prognosis. Albinism is characterized by a non-progressive vision decline and no effect on brain capabilities. Treatment primarily involves avoiding UV exposure and routine eye exams to control amblyopia. Assessments for low vision and appropriate recommendations to support children at school are also beneficial.

### 2.4. X-Linked Juvenile Retinoschisis

X-linked juvenile retinoschisis (XLRS) is the leading hereditary condition causing a form of maculopathy in males during childhood. It accounts for approximately 5% of all inherited retinal disorders with progressive onset in early life. The global prevalence of XLRS is estimated to range between 1 in 5000 and 1 in 25,000 males [96].

XLRS is a disorder due to mutations in the *RS1* gene. This gene encodes retinoschisin, an extracellular binding protein. XLRS follows an X-linked inheritance. In women, both copies of the gene must be mutated to manifest the disorder. Since it is rare for females to have two altered copies of the gene, X-linked recessive conditions are much more common in males. One feature of X-linked inheritance is that men cannot transfer X-linked mutations to boys. However, if lyonization occurs, some women may exhibit clinical manifestations of the disease [97].

The effect of specific mutations on the retinoschisin protein structure has been evaluated in several studies. Conservative *RS1* missense mutations, which preserve the charge and size of the residue, are likely to cause minimal disruption to the protein structure and are generally classified as “less severe”. On the other hand, mutations that result in significant structural changes or substantially reduce retinoschisin production are considered “more severe”. These include mutations that add or delete a cysteine residue, disrupting important disulfide bonds critical for the protein’s tertiary structure. Frameshift mutations can modify all subsequent amino acid sequences, whereas splice site mutations disrupt the coding process at the intron–exon boundary. Nonsense mutations typically cause early termination of protein synthesis, leading to swift degradation of the retinoschisin, as shown by biochemical studies [98].

**Figure 4:**
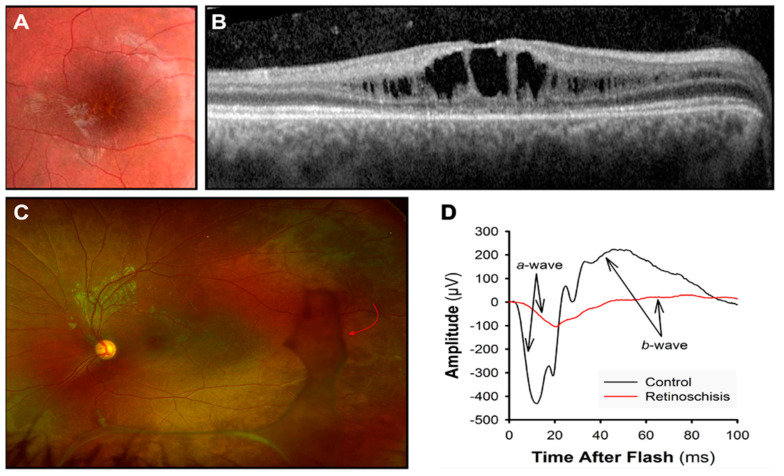
X-linked retinoschisis. (**A**) “Spoke-wheel” pattern on a fundus image of the macula. (**B**) Dark spaces showing the cystic cavities at the OCT. (**C**) In an ultra-wide-field retinal image, vitreous veils (red arrow) are visible in the peripheral retina. (**D**) ERG response is reduced: a-wave photoreceptor response and b-wave bipolar cell response.

Retinoschisin is primarily present in the inner segments of photoreceptors [99,100]. It is secreted as a disulfide-linked octamer that plays a crucial role in maintaining the structural integrity of the retina [101]. When functional retinoschisin is lost, cystic cavities form between the outer plexiform layer (OPL) and outer nuclear layer (ONL) of the retina [102,103]. These cystic changes are often observed during ophthalmoscopy as a “spoke-wheel” shape in the macula (Figure 4). These structural alterations are linked to a progressive decline in visual acuity [104]. In a mouse model, photoreceptor degeneration began early, progressively damaging the OPL, where photoreceptors synapse with bipolar cells in the inner nuclear layer (INL) [105].

Vision loss is the most prevalent symptom in XLRS. Additional frequently observed features in affected boys include strabismus, nystagmus, amblyopia, significant refractive errors, and visual field abnormalities.

Ophthalmoscopy is the primary diagnostic tool, although the fundus may appear normal in very young children. A thorough fundus exam, including the peripheral retina, is recommended to detect peripherical schisis and vitreous veils (Figure 4). These findings are markers of a higher risk for retinal detachment.

ERG waveforms can reveal bipolar cell impairment. Bipolar cell activity reflects in the cornea-positive b-wave, which is reduced compared to the photoreceptor activity (Figure 4D). This results in a “negative ERG” [96,106]. Nevertheless, more recent reports have also highlighted a photoreceptor contribution in retinoschisis. Additionally, with adaptive optics (AO) retinal imaging, cone photoreceptor anomalies have been demonstrated in boys with XLRS [107,108,109,110].

OCT helps in the detection of schisis cavities, identifying the characteristic splitting of the retinal layers, particularly in the inner retina. OCT also helps to assess the extension and the location of the cavities: it helps determine the size and precise location of the schisis cavities, such as in the macular or peripheral retina (Figure 4B).

Incomplete congenital stationary night blindness (CSNB) and XLRS share similar features, such as electronegative waveforms on ERG and reduced dark-adapted responses. However, mutations in CSNB-associated genes result in a less progressive course of symptoms.

Macular schisis findings should be differentiated from other forms of macular schisis, such as those linked to myopia or cystoid macular edema. The latter can occur in association with various conditions, including uveitis, retinal vein occlusion, and diabetes.

Patients with XLRS should undergo routine evaluations by a retinal specialist and a retinal surgeon. Individuals with high refractive errors are at greater risk for complications such as retinal hemorrhage and detachment; therefore, participation in contact sports is strongly discouraged.

Low-vision aids, including electronic, computer-based, and optical devices, may be beneficial for patients with reduced visual acuity. Orientation and mobility training, often provided by community resources, is especially valuable for younger patients.

Visual acuity commonly declines during childhood and adolescence but tends to stabilize in adulthood, until a significant deterioration typically occurs in the fifth or sixth decade of life. Severe complications, including retinal layer splitting, retinal detachment, or vitreous hemorrhage, may arise and further compromise vision or lead to blindness [111].

Currently, there are no effective treatments for XLRS. Gene therapy is considered the most valid approach, and there is significant global anticipation regarding its potential efficacy.

In preclinical studies, gene therapy demonstrated success in a mouse model [100]. In the RS1-/y knockout mouse, intravitreal administration of an adeno-associated virus (AAV) vector carrying the *RS1* gene slowed retinal degeneration and improved ERG outcomes significantly [100,105,112,113]. However, results from two Phase I/IIa clinical trials [114,115] using AAV8-RS1 gene therapy revealed substantial inflammatory responses and an effective outcome only in one patient. While innovative, these trials did not produce significant improvements in retinal function, as evaluated by VA, perimetry, OCT, or electrophysiology. Furthermore, intraocular inflammation, including anterior and/or posterior uveitis, was observed in both studies. However, these inflammatory responses were largely managed with systemic steroids. The discrepancy between the effectiveness observed in mice and the limited success in humans remains unexplained. This disparity may stem from fundamental differences between the RS1 knockout model and the common mutations in human XLRS. Unlike the knockout model, most human mutations lead to the production of mutant RS1 protein rather than a complete absence of the protein [116].

Recent studies [108,110,117,118] utilizing ERG and AO cellular imaging in boys with XLRS suggest that future therapeutic strategies might benefit from focusing on phototransduction pathways. The molecular phototransduction cascade, which shares similarities with signaling pathways in bipolar cells, offers multiple potential pharmacological targets. Further investigation of the relationship between structural and functional abnormalities could aid in identifying and refining therapeutic targets.

Interestingly, carbonic anhydrase inhibitors (CAIs) have shown measurable benefits in XLRS, although the mechanisms behind their effectiveness remain unclear [119]. CAI treatment has been associated with reduced macular thickness, improved visual acuity, and improved macular function, as evidenced by psychophysical and ERG measurements [120]. These agents can be administered topically (e.g., dorzolamide) or systemically (e.g., acetazolamide). While CAIs do not directly address the underlying pathophysiology of XLRS, they act by inhibiting the conversion of carbon dioxide and water into carbonic acid, which subsequently generates bicarbonate.

The proposed mechanism involves facilitating fluid transport out of the retina, reducing cystic space volume, and potentially promoting cellular adhesion [121,122,123]. This may be achieved through increased acidification of the subretinal space, which results in enhancing fluid movement to the choroid [124,125].

## 3. Conclusions and Future Directions

The future of treatment for pediatric macular disorders lies in a combination of advanced molecular therapies, innovative drug delivery systems, and precision medicine approaches. Gene therapy is at the forefront, with ongoing research into AAV-mediated gene delivery to correct pathogenic mutations, as exemplified by promising trials targeting *ABCA4* and *BEST1* mutations. Complementing this, emerging genome-editing technologies such as CRISPR/Cas9 offer the potential for precise correction of genetic defects at the DNA level. Pharmacological approaches aimed at modulating disease pathways, including inhibitors of toxic metabolite accumulation and antioxidants to mitigate oxidative stress, are under active investigation. Additionally, the development of cell-based therapies, such as stem cell-derived retinal pigment epithelial cell transplantation, seeks to restore structural and functional integrity to the degenerating retina. Advances in nanotechnology are also enabling the creation of targeted drug delivery systems that enhance therapeutic efficacy while minimizing systemic side effects. Personalized medicine, driven by whole-genome sequencing and biomarker discovery, promises to optimize treatment regimens tailored to the genetic and phenotypic profiles of individual patients. As these therapies progress from bench to bedside, collaborative efforts between researchers, clinicians, and industry will be pivotal in translating these innovations into effective, accessible treatments for children affected by macular disorders.

## Figures and Tables

**Table 1:** Juvenile macular disorders: molecular mechanisms, clinical aspects, therapeutic approaches.

Aspect	Stargardt Disease	Best Vitelliform Macular Dystrophy	X-Linked Retinoschisis
Mechanism	Mutations in the ABCA4 gene leading to dysfunctional protein involved in the visual cycle and lipid transport.	Mutations in the BEST1 gene affecting ion transport in retinal pigment epithelial cells.	Mutations in the RS1 gene encoding retinoschisin, essential for retinal structure and synaptic function.
Inheritance	Autosomal recessive inheritance.	Autosomal dominant and recessive inheritance.	X-linked inheritance.
Clinical Stages	Early: Flecks at the macula, reduced central vision. Late: Bull’s eye maculopathy, progressive vision loss.	Early: Yellowish deposits (vitelliform lesion). Late: Atrophy and scarring leading to vision loss.	Early: Foveal schisis with cystic spaces. Late: Peripheral retinal degeneration, potential retinal detachment.
Pharmacologic Strategies	Experimental: Vitamin A modification, gene therapy trials targeting ABCA4.	Limited to supportive care; gene therapy targeting BEST1 under investigation.	Carbonic anhydrase inhibitors to reduce cystic spaces; gene therapy under research.
Recent Therapeutic Advances	Gene editing using CRISPR/Cas9; stem cell-based therapies showing potential.	Novel gene replacement strategies and antisense oligonucleotides in preclinical stages.	Advances in adeno-associated virus (AAV) vectors for gene delivery; early-stage clinical trials ongoing.
